# Pseudoxanthoma elasticum, a difficult diagnostic in patient with dark skin

**DOI:** 10.1002/ski2.385

**Published:** 2024-04-11

**Authors:** Carla Fassanaro, Morgane Stichelbout, Marc Lambert, Frédéric Dezoteux, Delphine Staumont‐Sallé, Marie Boileau

**Affiliations:** ^1^ Department of Dermatology CHU Lille University Lille Lille France; ^2^ CHU Lille Centre de Biologie Pathologie Pierre‐Marie Degand Lille France; ^3^ Department of Internal Medicine CHU Lille University Lille Lille France; ^4^ CHU Lille University Lille U1286 Inserm INFINITE ‐ Institue for Translationnal Research in Inflammation Lille France

## Abstract

Pseudoxanthoma elasticum is a genetic metabolic disease which leads to ectopic mineralisation in the elastic tissues of the skin, eyes and blood vessels. The clinical signs are small yellow or normal skin‐coloured papules on the nape of the neck and lateral sides of the neck, as well as in flexural areas and periumbilical region. The skin becomes loose and wrinkled. The diagnosis on dark skin is particularly difficult. The dermatologist evokes the diagnosis and refers the patient to specialists in order to detect complications. We propose here a practical case study.

## Graphical abstract

Pseudoxanthoma elasticum (PXE) is a genetic metabolic disease which leads to ectopic mineralisation of the elastic tissues in the skin, eyes and blood vessels. The clinical signs are small yellow or normal skin‐coloured papules on the nape and lateral sides of the neck, as well as in flexural areas and periumbilical region. The skin becomes loose and wrinkled. The diagnosis on dark skin is particularly difficult. The dermatologist evokes the diagnosis and refers the patient to specialists in order to detect complications. We propose here a practical case study.

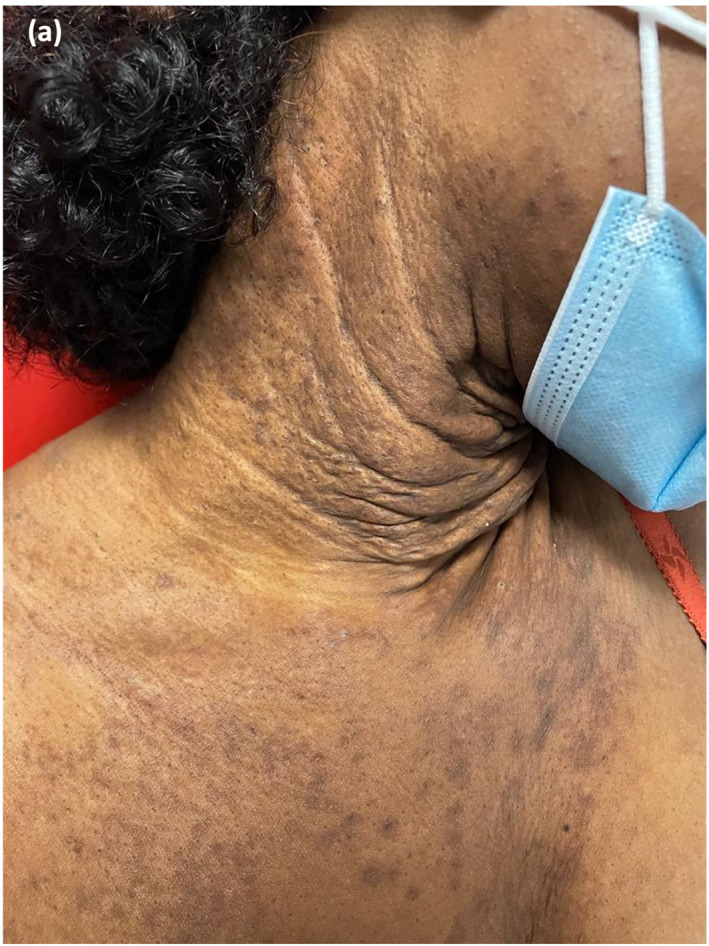

## 1 INTRODUCTION

Pseudoxanthoma elasticum (PXE) is a genetic metabolic disease with autosomal recessive inheritance caused by mutations in the *ABCC6* gene.^1^ The lack of functional ABCC6 protein leads to ectopic mineralisation in the elastic tissues of the skin, eyes and blood vessels.^2^ The rarity of the pathology and the discretion of the dermatological signs can lead to a misdiagnosis. Experience in dark skin is limited. This case is a great illustration of a clinical presentation that can be discrete. We propose a focus of the clinical presentation, diagnostic criteria and management based on a case report.

## 2 OBSERVATION

An 18‐year‐old female patient from Guinea (phototype V) with no previous medical history was seen in consultation in our department for a pruritic dermatosis evolving since the age of 10. She had a family history of pruritic dermatitis diagnosed as eczema in her younger sister which started at the same age.

**FIGURE 1:**
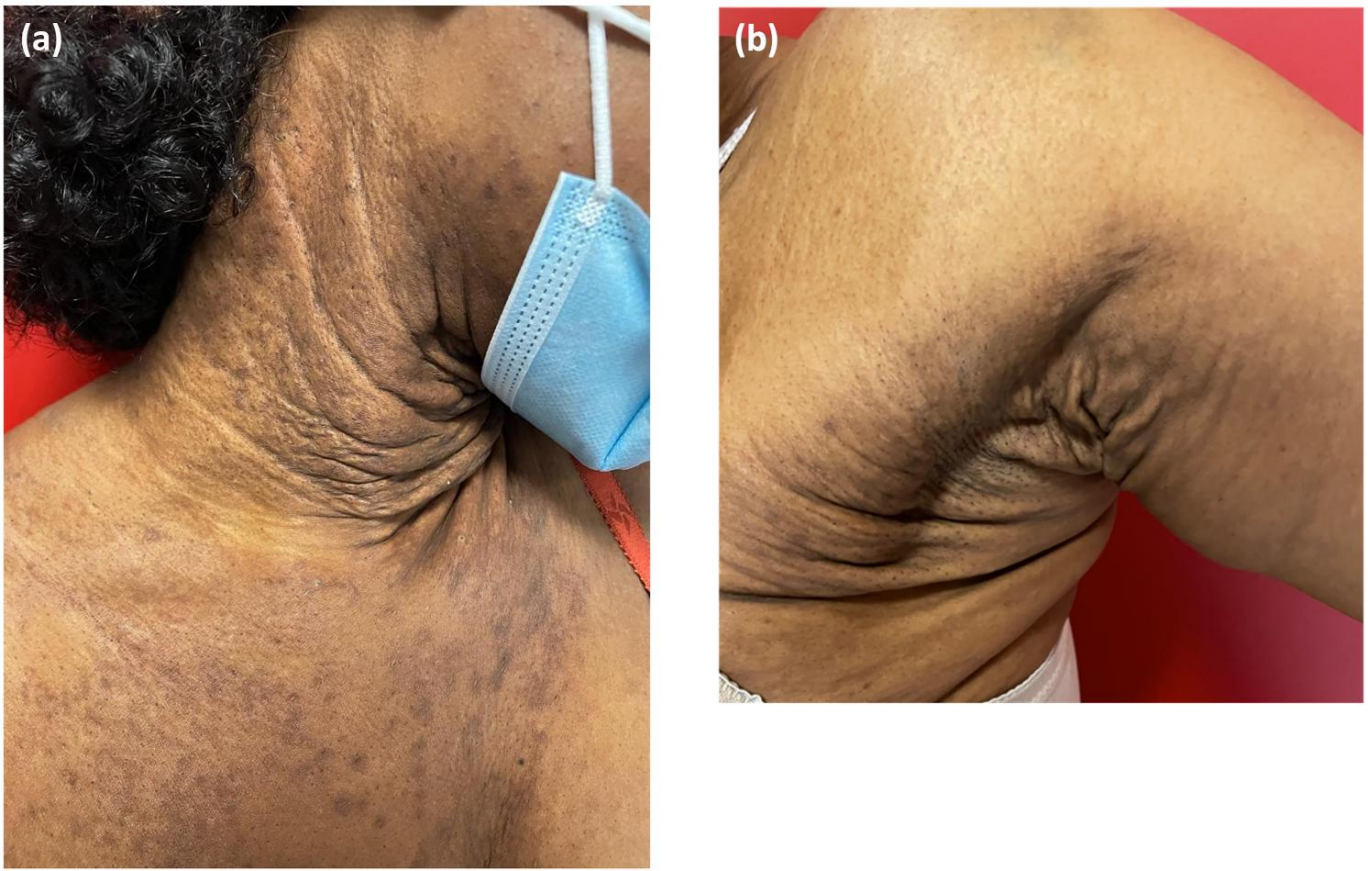
(a) A yellowish pigmented squared aspect of the neck initially confused with an old lichenified localization of atopic dermatitis; (b) aspect of axillary skin surplus leading to question the diagnosis.

Pruritus predominated on the neck, elbow creases, axillae and abdomen. A squared and lichenified aspect of the neck was noted as well as pigmented lesions of the neck, the axillary folds and the abdomen (Figure 1a,b).

Due to the pruritic and lichenified character of the skin and by argument of frequency, the diagnosis of atopic dermatitis was retained. The EASI score at the first presentation evaluated by the clinician was 10. A high potency topical corticosteroid once a day was started.

One year later, the initial diagnosis of severe lichenified atopic dermatitis was questioned, given the cortico‐resistance. Then, the hypotheses were a lichen planus, a Dowling Degos syndrome or a genetic disease of elastic tissues.

**FIGURE 2:**
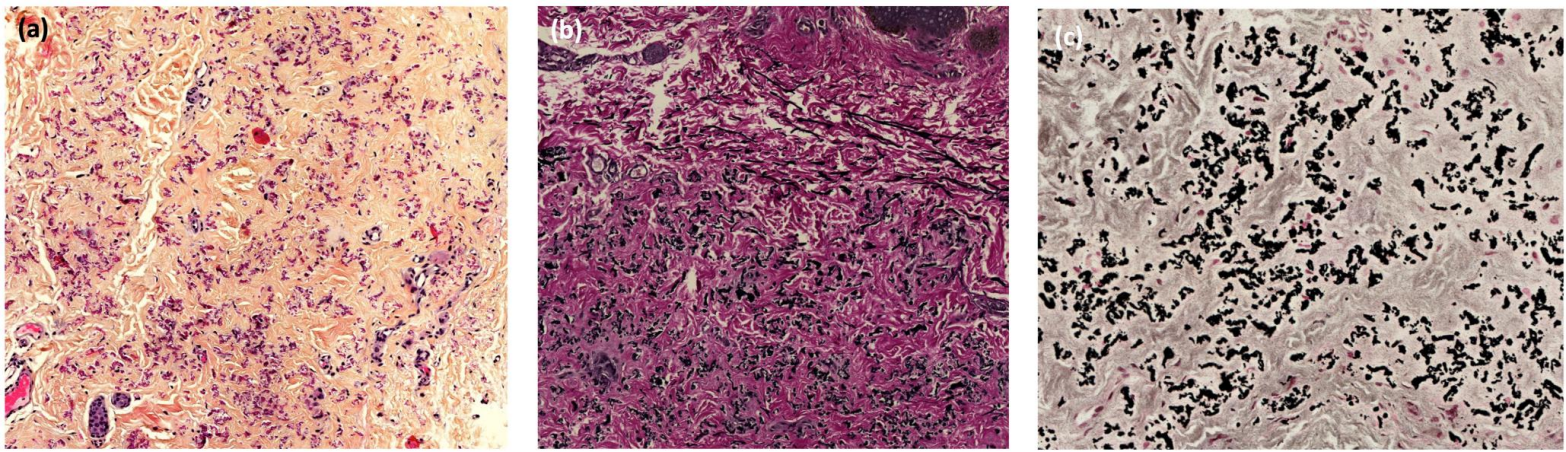
(a) HES stain x200, Lille University Hospital; (b) Orcein stain x200—presence of thick and fragmented elastic fibres; (c) Von Kossa stain x200—presence of calcium deposits.

Skin biopsies performed on the neck, axillary folds and abdomen revealed significant histological elastotic remodelling of the middle and deep dermis, thick and fragmented elastic fibres (orcein staining) and numerous calcium deposits in Von Kossa staining (Figure 2).

The diagnosis of PXE was made. An ophthalmologic and vascular evaluation was scheduled and will be repeated one a year. Arterial ultrasound of the upper and lower limbs showed no thrombosis and ophtalmologist's examination was normal. The genetic study confirmed PXE with double heterozygosity in the ABCC6 gene. Two variants were identified: p.Arg419gln and p.Ser245del. A CO^2^ laser treatment was proposed to treat cervical skin, but our patient didn't want to try it. Topical corticosteroid was stopped to avoid potential side effects such as usual skin atrophy. Prolonged use of topical corticosteroid can also lead to cortico‐induced folliculitis in dark‐skinned patients, with unaesthetic iatrogenic depigmentation.

## 3 DISCUSSION

Pseudoxanthoma elasticum is a rare disease (prevalence between 1/100,000 and 1/25,000 in the general population) with slight female predominance.^1^ The extracutaneous, ophthalmological and vascular disorders are the main causes of the severity of the disease. An early diagnosis is necessary to prevent complications.

The diagnosis may be difficult and the dermatologist has a central role in the detection of this genetic pathology. Indeed, especially in childhood and adolescence, cutaneous signs are often very slight. The diagnosis is based on a combination of clinical (small yellow papules progressively coalescent into reticulated plaques, involvement of neck and folds), histological (damages of elastic tissues) and/or molecular evidence.^3^ There are no widely accepted international recommendations for the clinical and genetic diagnosis of PXE. A classification was proposed in 2010 by *Plomp and al*.^3^ Table 1.

**TABLE 1:** Revised diagnostic criteria for PXE.^3^

Major diagnostic criteria	
Skin	a. Yellowish papules and/or plaques on the lateral side of the neck and/or flexural areas of the body; or
	b. Increase of morphologically altered elastin with fragmentation, clumping and calcification of elastic fibres in a skin biopsy taken from clinically affected skin
Eye	a. Orange skin of the retina; or
	b. One or more angioid streaks (ASs), each at least as long as one disk diameter. When in doubt, fluorescein or indocyanine green angiography of the fundus is needed for confirmation.
Genetics	a. A pathogenic mutation of both alleles of the ABCC6 gene; or
	b. A first‐degree relative (parent, sib, child) who meets independently the diagnostic criteria for definitive PXE
Minor diagnostic criteria	
Eye	a. One AS shorter than one disk diameter; or
	b. One or more ‘comets’ in the retina; or
	c. One or more ‘wing signs’ in the retina
Genetics	A pathogenic mutation of one allele of the ABCC6 gene
Requirements for the diagnosis of PXE	
Definitive diagnossis	The presence of two (or more) major criteria not belonging to the same (skin, eye, genetic) category
Probable diagnosis	The presence of two major eye or two major skin criteria, or the presence of one major criterion and one or more minor criteria not belonging to the same category as the major criterion
Possible diagnosis	The presence of a single major criterion, or the presence of one or more minor criteria

Definitive diagnosis is based on the presence of two major criteria not belonging to the same category (skin, eye, genetic).

In darker phototype patients the diagnosis is even more difficult.^4^ Experience of PXE on black skin is limited. There are very few cases described in the literature. The usually yellowish papules of the folds are darker on black skin and evolves into brownish plaque with a cartoon‐like consistency in usual locations of PXE (neck, armpits, elbow folds).^5^ Differential diagnoses are intense solar elastosis of the neck in the elderly, cutis laxa, *β*‐thalassaemia and sickle cell disease.^6^ In our patient, skin lesions mimicking lichenification in the folds, associated with intense pruritus and a family past history of pruritic dermatosis, led us to the first diagnosis of atopic dermatitis. Nevertheless, inefficacy of topical corticosteroids justified the histological analysis of skin lesions, which allowed us to correct the diagnosis. We were not able to examine the patient's sister to confirm or not whether she had also signs of PXE.

Once the diagnosis has been made, the dermatologist can play an essential role in coordinating the prevention of complications.

Skin treatments are essentially symptomatic.^7^ Cosmetic surgery usually consists of lower subcutaneous rhytidectomy and neck skin lift performed through a standard preauricular facelift incision with postauricular extension and transverse extension into the hairline with excellent results and minimal complications.^6^ Chin folds and wrinkles can be temporarily treated with collagen injections.^8^ The CO^2^ laser was used on one patient with a successful result.^9^ Cosmetic surgery or laser treatment may be proposed in case of major discomfort and impairment of quality of life. Ophthalmologic and cardiac follow‐up is necessary but the frequency is not defined.^10^ In our patient's case, follow‐up will consist of an annual ophthalmological (with a fundus) and cardiological examination. Reducing cardiovascular risk factors is essential to slow the onset of complications.^1^ This includes smoking cessation, a healthy, Mediterranean diet, physical activity, preferably on a regular basis, weight control (body mass index <25 kg/m^2^), taking medications as prescribed, and the treatment of comorbidities, such as arterial hypertension (AHT) and diabetes.^7^ An ECG and Doppler ultrasound should be performed at diagnosis, and then on a regular basis. Japanese guidelines recommend a screening by imaging techniques such as myocardial scintigraphy and coronary artery assessment by computed tomography (CT) or magnetic resonance imaging (MRI) in patients with severe angina.^11^ The molecular diagnosis of PXE is established in a patient by the presence of mutations in the ABCC6 gene identified by molecular genetic testing. The genetic study is not a routine basis but is made in scientific laboratories expert centres. More than 300 variants have been identified.^1^ Mutations are over‐represented in some populations. The R1141X mutation is the most common one detected in Caucasians.^12^ In North Africans, p.Arg518Gln, p.Glu1400Lys and p.Arg1314Trp mutations were over represented.^13^ Some authors have also found a correlation between symptom severity symptoms and ethnic origin. The study by Legrand et al shows more severe occular damage in Caucasians than in North Africans.^13^ Molecular testing is not necessary if clinical cutaneous and ophthalmological criteria are met. Genetic counselling should be offered for siblings. Indeed, PXE is transmitted according to a Mendelian autosomal recessive inheritance, with a 25% risk of recurrence in siblings.^1^

Furthermore, the question of prenatal diagnosis is open to debate, given that this is only exceptionally life‐threatening.^14^ Prenatal diagnosis is not currently recommended in France.^15^

## 4 CONCLUSION

In conclusion, the diagnosis of PXE is difficult, especially in patients with a dark phototype, but the characteristic clinical presentation should retain the interest of the dermatologist who plays a central role in screening and referring the patient to a team of specialists for the management of potential complications arising from this genetic pathology.

## CONFLICT OF INTEREST STATEMENT

None to declare.

## AUTHOR CONTRIBUTIONS

**Carla Fassanaro**: Writing – original draft (equal). **Morgane Stichelbout**: Writing – original draft (equal). **Marc Lambert**: Visualisation (equal). **Frederic Dezoteux**: Writing – original draft (equal). **Delphine Staumont‐Salle**: Writing – original draft (equal). **Marie Boileau**: Conceptualisation (equal).

## ETHICS STATEMENT

According to standard procedure and the Jardé Law (March 2012) on the publication of retrospective data, the patient of the case report expressed her nonopposition to the use of her anonymised medical data.

## Data Availability

The data underlying this article will be shared on reasonable request to the corresponding author.
